# A multiline fitting method for measuring ethylene concentration based on WMS-2f/1f

**DOI:** 10.1038/s41598-023-42398-2

**Published:** 2023-09-15

**Authors:** W. F. Wang, B. Yang, H. F. Liu, L. F. Ren, D. He, X. C. Zhao, J. Li

**Affiliations:** 1 School of Safety Science and Engineering, Xi’an University of Science and Technology, Xi’an, 710054 Shaanxi China; 2 Key Laboratory of Mine and Disaster Prevention and Control of Ministry of Education, Xi’an University of Science and Technology, Xi’an, 710054 Shaanxi People’s Republic of China

**Keywords:** Engineering, Electrical and electronic engineering

## Abstract

Coal spontaneous combustion risk assessment is a global technical challenge for the sustainable development of deep mining technology, and C_2_H_4_ is a key indicator for early warning of coal spontaneous combustion. Tunable diode laser absorption spectroscopy (TDLAS) has the advantages of high selectivity, high sensitivity, high accuracy and real-time on-line measurement, and it can detect multiple gases simultaneously, so it has significant advantages in the accurate detection of coal spontaneous combustion indicator gases. To address the problem of cross-interference between the near-infrared absorption lines of CH_4_ and C_2_H_4_, which are the indicator gases of spontaneous combustion in coal, a multi-line fitting method was proposed in this study to calibrate the concentration of C_2_H_4_. The high-precision Environics2000 automatic standard gas dispenser from the United States, which has a built-in CPU computer control and data control and processing system, was used. Its gas concentration accuracy: ± 1.0%, gas flow accuracy: ± 1.0%, gas repeatability accuracy: ± 1.0%, flow linearity accuracy: ± 0.5%, and inlet operating pressure: minimum 10 psig (0.67 bar) ~ 75 psig (5.04 bar). The measured and simulated WMS-2f/1f signals were multilinearly fitted using a multilinear fitting algorithm and wavelength modulation spectroscopy (WMS), and the measurement of C_2_H_4_ concentration was achieved based on the extracted spectral line information. The results show that the maximum relative error of C_2_H_4_ concentration measurement is 2.40%, which is 54% lower than that of the conventional 2f peak measurement method, thus demonstrating the effectiveness of the multilinear fitting algorithm in the inversion of C_2_H_4_ concentration under the interference of absorption lines. In addition, this study has far-reaching implications for the application of TDLAS technology in the accurate detection of coal spontaneous combustion indicator gases.

## Introduction

Spontaneous coal combustion disasters are found throughout the world, which have become a global issue since they cause tremendous loss of coal resources and environmental pollution every year, seriously threatening the ecological environment and human health^1^. In China, coal fire disaster is one of the five major disasters that threaten the safety of coal mines, which is extremely serious as over 90% of the coal seams are prone to spontaneous combustion^2,3^. During monitoring and early warning of spontaneous coal combustion, the physicochemical characteristics of coal and rock and their variation trends over the combustion process are explored by utilizing the indicator gas or temperature detection technology^4,5^. Methods used globally for detecting the indicator gas and temperature of spontaneous coal combustion mainly include: the indicator gas sampling and analysis^6^, the tracer gas leak detection^7^, the odor detection and analysis^8^ and the temperature detection^9^. So far, the above methods have exerted excellent roles in the prediction and forecasting of spontaneous coal combustion. Nevertheless, with the progress of coal mining technologies and the increasing complexity of ventilation systems^10^, great challenges have been encountered in the combustion monitoring and early warning^11^. C_2_H_4_ gas appears in the pyrolysis stage (110–150°C) of spontaneous coal combustion^12^, whose concentration range is 0–500 ppm. Therefore, its appearance has important significance and value for the early risk identification and early warning of spontaneous coal combustion.

As a novel method of high-sensitivity gas detection, the tunable diode laser absorption spectroscopy (TDLAS) technology has the advantages of non-contact, high selectivity, high sensitivity, rapidity, high efficiency, long calibration period and dynamic detection^13,14^, which allows simultaneous detection of multiple gases^15,16^. There have been extensive studies concerning the TDLAS gas detection both in China as well as other countries^17–19^. Given the importance of ethylene in industry, agriculture and environmental protection, its measurement based on TDLAS has received growing attention in recent years. Utilizing a 3,266 nm interband cascade laser (ICL) as the light source and a hollow wave guide (HWG) as the gas chamber, J. Li et al. developed a TDLAS-based sensor platform, through which the sensitivity of C_2_H_4_ detection was enhanced by adopting the wavelength modulation spectroscopy with second harmonic detection (WMS-2f)^20^. J. Kathirvelan et al. fabricated an ethylene sensor based on infrared radiation. Since ethylene could absorb the infrared spectrum at 10.6 µm, its concentration in fruit samples decreased persistently from 59 to 5 ppm. The sensor had sensitivity values of 3.3 ± 0.2%, exhibiting good reproducibility^21^. After exploring the absorption line characteristics of ethylene in the near-infrared band, W.D. Pan et al. developed a TDLAS detection system based on white cell structure by selecting the absorption peak near 1626.8 nm as the detection line. By exploiting this system along with the WMS-2f technique, they measured the ethylene gases with volume fractions of 20–1200 ppmv, and estimated the lower detection limit for the system to be approximately 10 ppmv^22^. Wavelength modulated spectroscopy (WMS) techniques using tunable diode laser absorption spectroscopy (TDLAS) have been used to measure ethane concentrations in exhaled gases by scanning ethane non-absorption lines at 2986.7 cm^-1^ and 2988.2 cm^-1^, allowing high sensitivity detection in both ethane concentration ranges. A highly sensitive mid-infrared dual range real-time trace sensor has been developed for the detection of ethane in exhaled gases, where the sensor employs an interband cascade laser (ICL) in continuous wave (CW) mode with an effective optical absorption length of 76.3 m in a multi-channel gas cell (MPGC)^23–27^. A compact near-infrared (NIR) laser-based detection system for trace methane (CH4) has been investigated by J.M. Dang et al. The detection system relies primarily on a 2334 nm distributed feedback (DFB) fibre laser. To improve the sensitivity of the system, they used a parallel dense spot mode multi-channel gas cell (MGC) with an effective absorption path length of 41.5 m and a self-calibration method based on direct absorption spectroscopy (DAS) calibrated wavelength modulation spectroscopy (WMS) technique, which solves the problem of the need for additional concentration calibration in conventional WMS techniques and improves the accuracy and stability of the system. With Allan deviation analysis processing, the 1s measurement accuracy can reach 0.61 ppmv for DAS and 0.16 ppmv for WMS, which can be further reduced to 0.11 ppmv for DAS and 0.03 ppmv for WMS by averaging over 80 s and 50 s.^28–30^. A second harmonic phase method based on wavelength modulated spectroscopy (WMS-theta(2f)), which is mainly used for trace gas detection, was proposed by C.G. Zhu et al. WMS-theta(2f) has the advantage of being background-free and unaffected by fluctuations in light intensity. Its detection sensitivity is 1-2 orders of magnitude higher than that of the first harmonic phase angle detection method. Furthermore, there is room for further improvement in sensitivity by optimising the linearity of the laser's light intensity modulation, validating the feasibility of the WMS-theta (2f) method^31,32^.

In summary, although the TDLAS technology has achieved certain results in the ethylene gas detection, the detection of trace C_2_H_4_ in the spontaneous coal combustion scenario has scarcely been explored. Since the spontaneous coal combustion indicator gases, C_2_H_2_, CH_4_ and C_2_H_4_, are hydrocarbon gases sharing similar molecular structures, cross-interference is present among the absorption spectral lines, causing a low accuracy of trace C_2_H_4_ detection. Aiming at the problem of cross-interference between the near-infrared absorption spectra of CH_4_ and C_2_H_4_, this paper proposes a multilinear fitting method for calibrating the C_2_H_4_ concentration. By using the multilinear fitting algorithm and the WMS technology, the multilinear fitting is carried out on the measured and simulated WMS-2f/1f signals, so as to realize the high-precision measurement of the C_2_H_4_ concentration. This paper provides a theoretical basis for the effective separation and concentration inversion of the cross-mixed spectral lines of multi-component gases in spontaneous coal combustion.

## WMS technique

The WMS^33^ exploits the tunable characteristic of wavelength output by tunable diode laser to lock the gas laser output wavelength to the peak center of target gas’s optimal absorption line, and uses sawtooth signal of a certain frequency to modulate the laser output wavelength, which can thus obtain the absorption line distribution of the target gas. Finally, it derives the gas concentration through inversion calculation. The current major method is WMS-2f/1f model^34^, which achieves the calibration-free detection of target gases by simulating the 2f signal. Based on the WMS, the influence of incident light intensity fluctuation on the detection accuracy is eliminated by normalizing the 2f signal by the 1f signal. If the eigen parameters including the incident intensity and pressure broadening of gas molecules are determined, the target gas concentration and absolute temperature can be directly measured without calibration by gas sample under actual conditions, thus achieving calibration-free detection. This method is suitable for harmonic signal detection systems where the wavelength and intensity modulations coexist.

Through normalization of the 2f signal by the detected 1f signal of gas absorption spectrum, the influences of factors like laser source, gas absorption cell and photoelectric detector on the laser intensity are eliminated. Thus, the TDLAS gas detection system measures the 1f and 2f signals of gas absorption lines simultaneously, and employs the 1f signal to normalize the 2f signal. The central peak of the normalized 2f signal absorption line is^35,36^

1 $$ C_{{2{\text{f}}/1{\text{f}}}} (\nu_{0} ) = - \frac{1}{{i_{0} }} \cdot \frac{{PS(T)C_{i} L}}{\pi }\int_{ - \pi }^{\pi } \varphi (\nu_{0} + a\cos \theta ) \cdot \cos 2\theta {\text{d}}\theta $$

Assume that the lineshape function $\phi (\upupsilon )$ of the target gas to be measured is unchanged, the C_2f/1f_ is directly proportional to the product of target gas concentration C_i_ and optical path L. C_2f/1f_ is merely a function of laser linear intensity modulating amplitude i_0_, constant a (the frequency modulation depth), gas pressure P, absorption line intensity S(T), gas concentration C_i_ and optical path L. θ = ωt, among ω = 2πfm is the angular frequency and t is the time. The eigen parameters of tunable diode laser can be obtained by experimental measurement. As a result, the target gas concentration can be detected without calibration as long as the P and L are determined.

## Experimental

### C_2_H_4_ absorption line analysis

**Figure 1:**
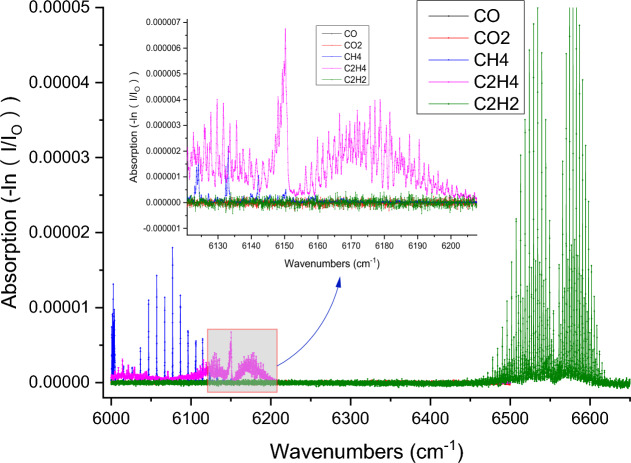
Absorption line distributions of spontaneous coal combustion multi-component gases in the near-infrared region.

For the TDLAS gas detection system, the near-infrared detection wavelength range of its photoelectric detector is generally 12,500–5555 cm^-1^. The infrared spectral characteristic parameters of C_2_H_4_ gas molecules in this paper are from the PNNL database on the website of the Northwest Pacific National Laboratory of the United States. Fig. 1 displays the absorption spectra of CO, CO_2_, CH_4_, C_2_H_4_ and C_2_H_2_ gases at a light range of 10 cm in the 6666–5988 cm^-1^ band, a temperature of 296 K and a pressure of 1 atm (CO, CO_2_, CH_4_, C_2_H_4_ and C_2_H_2_ gas concentrations are all 100 ppm). Evidently, the linear intensity of CO is overall weaker than that of CH_4_, C_2_H_4_ and C_2_H_2_, and the absorption lines of CH_4_, C_2_H_4_ and C_2_H_2_ are prominently overlapping. Within this band, the CO_2_ absorption line is comparatively weak. The cleanest and strongest absorption line was selected after comprehensively considering the overlapping phenomenon of various component gases and the absorption intensity of spectral lines. In general, the contents of spontaneous coal combustion multi-component gases CO_2_ and CH_4_ are in the 10^-4^–10^-2^ order-of-magnitude range, whereas the contents of CO, C_2_H_2_ and C_2_H_4_ are often in the 10^-6^ order-of-magnitude range. However, C_2_H_2_, CH_4_ and C_2_H_4_ are hydrocarbons gases sharing similar molecular structures, whose absorption spectral lines are subject to cross-interference, resulting in low sensitivity, precision and large error during the trace gas detection. The optimal absorption lines of CH_4_, C_2_H_4_ and C_2_H_2_ gases are 6046.97 cm^-1^, 6150.27 cm^-1^ and 6534.37 cm^-1^, respectively. Nevertheless, C_2_H_4_ has multiple absorption peaks in the wavelength range of 6153.85–6146.28 cm^-1^ and CH_4_ absorption lines are present at all the positions of these peaks, while are absent at 6148.76 cm^-1^. In addition, the CH_4_ concentration is normally far higher than the C_2_H_4_ concentration. Hence, the optimal absorption line of C_2_H_4_ gas was selected as 6,148.76 cm^-1^ (1626.343 nm) in order to minimize the interference of spectral line aliasing.

### Experimental system

**Figure 2:**
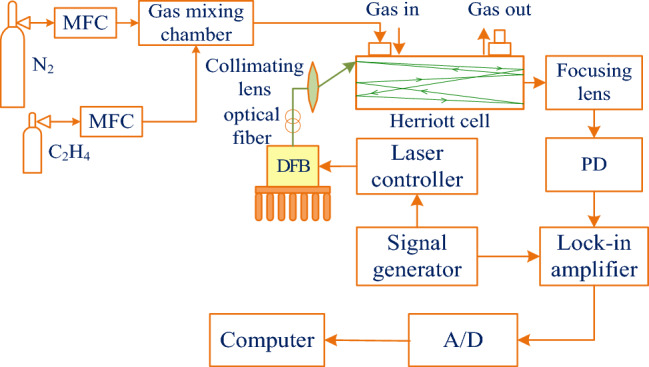
Experimental system for gas concentration calibration.

**Figure 3:**
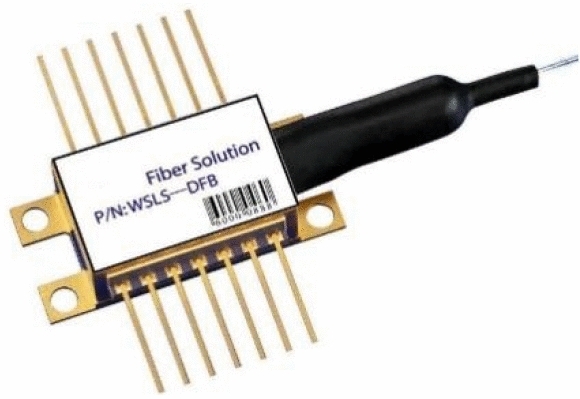
DFB laser.

**Figure 4:**
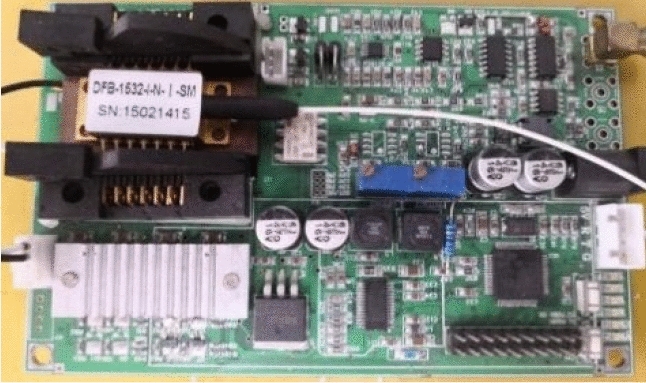
Laser current drive and temperature controller.

**Figure 5:**
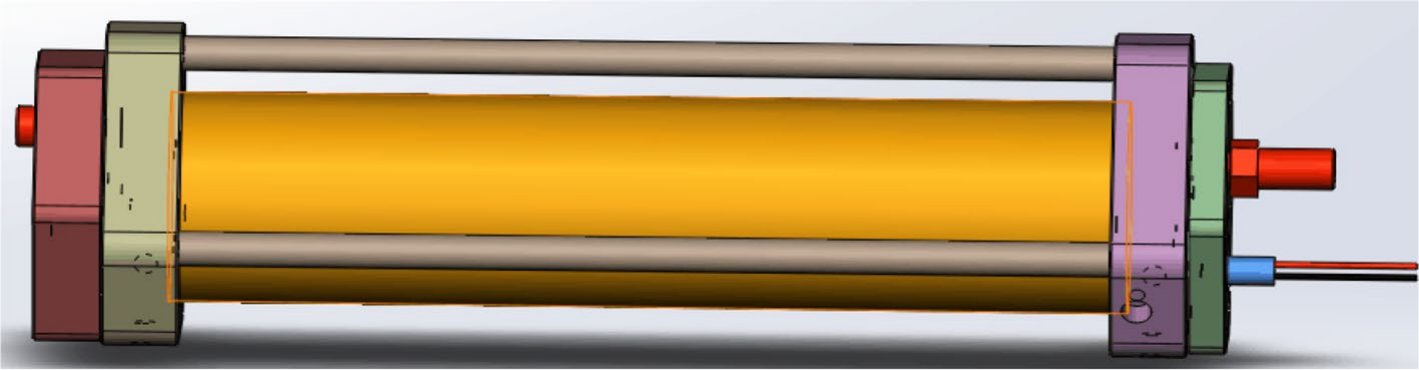
Herriott gas absorption cell.

**Figure 6:**
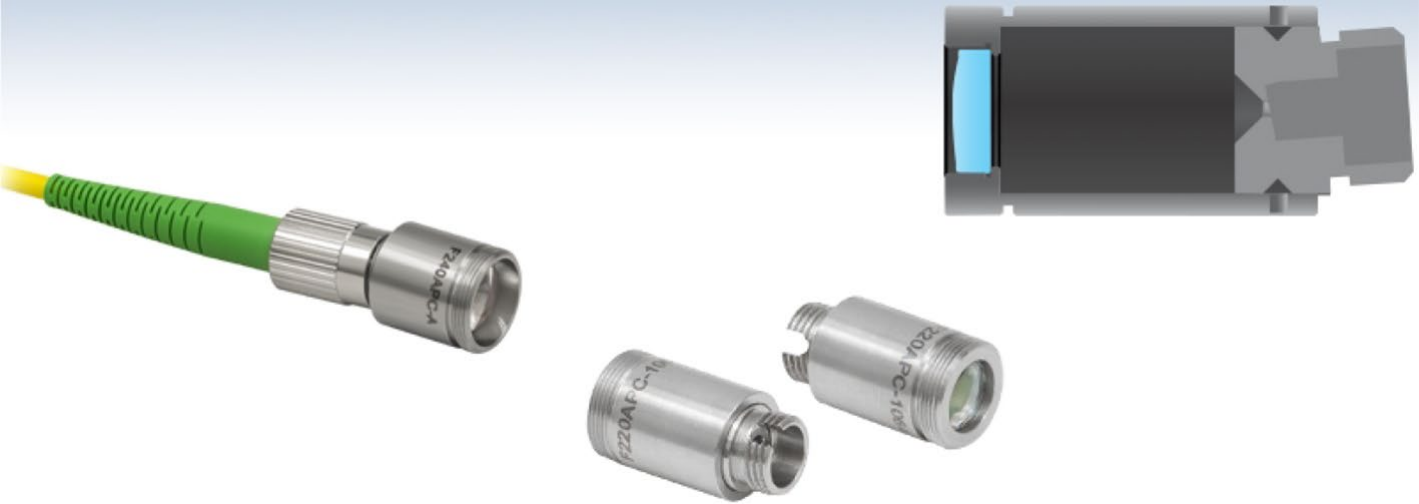
F240FC/APC-1550 Optical Fiber Collimator.

**Figure 7:**
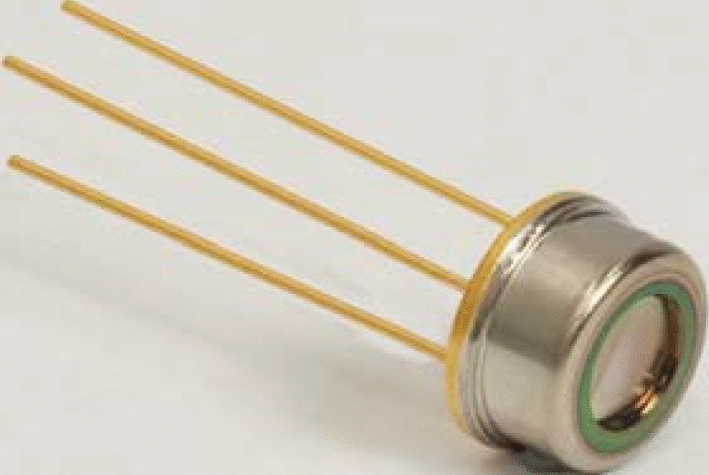
FGA10 Photodetector.

An experimental system for C_2_H_4_ concentration calibration was built by utilizing the Environics 2000 computorized multi component gas mixer. The main hardware of the experimental system in Fig. 2, such as DFB laser, laser current drive and temperature controller, Herriot gas absorption cell, optical fiber collimator and photoelectric detector, are shown in Figs. 3, 4, 5, 6, 7 respectively.The laboratory temperature is 26.4 °C, and the normal operating temperature range of the laser is 15 to 50 °C.

As shown in Fig. 3, DFB laser is a single-mode laser of Wuhan Liujiu Sensor Technology Co., Ltd. as the light source of C2H4. Peak Wavelength is 1627.002 nm, Speetral width is 0.097 nm, Pout is 5 mW, Ith is 12 mA, Iop is 50 mA, AMSR is 54.56 dB.

As shown in Fig. 4, the experimental system adopts LY-LIA-D0424 digital phase-locked amplifier (with preamplifier circuit). Its input channel can select voltage single terminal, differential, and current single terminal modes. The signal is amplified to the appropriate amplitude by programmable amplifier, and 50 Hz and 100 Hz notch filters are set to filter power frequency interference. The digital phase-locked amplifier supports an external input reference signal (square wave or sine wave). When the internal reference signal is used, the demodulation of two frequency signals (1–8f) can be realized, and the synchronous arbitrary waveform reference signal can be output.

As shown in Fig. 5, Herriot Gas Cell is mainly composed of gas chamber chamber, concave reflector, standard optical fiber connector, light detector, gas inlet and outlet, shock proof base, etc. It has a unique suspension optical path design, excellent vibration and temperature stability, effective optical path 14.4 m, beam diameter ≤ 3.5 mm, gas volume 0.84 L (1 atm), peripheral size 0.36 m (L) * 0.13 m (W) * 0.18 m (H), working pressure: 10–101,000 Pa, lens coating: oxide coating metal (reflectivity ≥ 98%), wavelength range: 0.2–12 μm.

As shown in Fig. 6, the light generated by DFB gas laser is Gaussian beam, which has a certain divergence angle, and beam diffusion will occur when it travels for a certain distance. In order to improve the quality of the laser beam and the detection accuracy, the TDLAS gas detection system needs to add a fiber collimator to convert the Gaussian beam into a parallel beam. This experimental scheme uses Thorlabs F240FC/APC-1550 fiber collimator to collimate the beam. The input FC/APC interface matches with the DFB gas laser fiber connector. The output aperture is 12 mm, the focal length is 8.18 mm, and the wavelength range is 1050–1620 nm. After the beam with 1550 nm is collimated, the spot radius is 0.8 mm, and the divergence angle is only 0.073°. The output lens is coated with an antireflection film, which greatly reduces the impact of reflected light.

As shown in Fig. 7, the experimental system uses Thorlabs' self balanced photodetector FGA10. FGA10 is an InGaAs photodiode with a rise time of 10 ns, a wavelength range of 800–1800 nm, and an effective area of photosensitive surface of ± 1 mm. FGA10 detector is used to reduce background noise through two matching large area InGaAs probes and an ultra-low noise transimpedance amplifier. There is a certain distance between the two probes, which is easy to realize the alignment with the light source.

The system has built-in CPU computer control and data control processing system, the first circuit range is 3000 mL/min, the second circuit range is 1000 mL/min, the third circuit range is 500 mL/min, the fourth circuit range is 200 mL/min, the fifth circuit range is 50 mL/min, the sixth circuit range is 20 mL/min, the seventh circuit range is 10 mL/min, and the eighth circuit range is 10 mL/min. The above ranges are using high purity nitrogen calibration test data, and provide 10-point calibration compensation parameters. Gas concentration accuracy: ±1.0%, gas flow accuracy: ±1.0%, gas repeatability accuracy: ±1.0%, flow accuracy: ±0.5%; inlet operating pressure: minimum 10 PSIG (68.9 kPa) to 75 PSIG (516.75 kPa). The conversion factors and computer coefficients for each component index gas of coal spontaneous combustion are shown in Table 1. The experimental system for gas concentration calibration was built using Environics 2000 high-precision gas calibration automatic gas distribution device.

**Table 1:** Conversion coefficients and calculation coefficients for each component index gas of coal spontaneous combustion.

Media	K-factor	Target concentration	Sample gas concentration	Concentration unit	Unit Conversions	Calculation factor	Flow/(mL/min)
CO	1.0000	10	1000	ppm	0.000001	1	10
CO_2_	0.7382	500	10,000	ppm	0.000001	0.996466069	50
CH_4_	0.7175	500	10,000	ppm	0.000001	0.996078159	50
C_2_H_4_	0.6000	20	1000	ppm	0.000001	0.999333777	20
C_2_H_2_	0.5829	20	1000	ppm	0.000001	0.999284952	20
O_2_	1.0000	21	100	%	0.01	1	210
N_2_	1.0000	78.8	100	%	0.01	1	632

The system used 8 gas mass flow controllers in combination, each of which was linearly interpolated with a 10-point calibration console, thereby improving the accuracy and reducing the controller nonlinearity. Within an equilibrium gas, 8 component gases could be mixed simultaneously, and the gas concentrations and the equilibrium gas flow were automatically calculated. Each component gas could be set via operational instructions, and the concentrations of resultant gases were of 10^-9^ order. The gas distribution mode of the experimental system was flow distribution, which was simple to use and easy to operate. In addition, it allowed the flow rate setting for each channel on the Windows interface via the touch screen, as well as display of the real-time feedback flow rates. During calibration experiments, a high-purity (99.999%) nitrogen was first introduced to purge the gas absorption cell, and thus the interference of residual gas inside the cell could be avoided. According to the volume of the cell cavity, the nitrogen purging time during the experiments was set at 5 min. Thereafter, the flow values were set for each channel of the Environics 2000 gas mixer, the intake valve was open, with the pressure reducing valve of gas under calibration, followed by initiation of the gas mixer. The calibration experiments started after the gas absorption cell was filled with the prepared gas under measurement, where the ventilation duration was 5 min.

## WMS-2f/1f-based multiline fitting algorithm

The WMS-(second harmonic value/first harmonic value) (hereinafter referred to as WMS-2f/1f) signal^37^ is decided by the integral absorbance of gas molecular absorption lines and their line shape function. Utilizing the multiline fitting algorithm, the detected and simulated WMS-2f/1f signals were subjected to multiline fitting, identifying the integral absorbance of absorption lines and their line shape function based on the fitted WMS-2f/1f signal. By exploiting the identified information, parameters like C_2_H_4_ gas concentration, temperature and pressure could be inverted.

**Figure 8:**
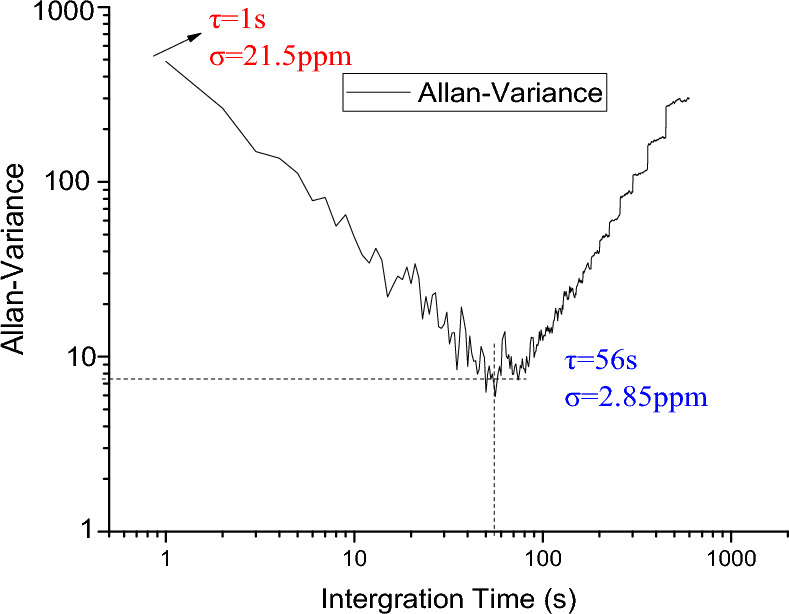
Allan variance evaluation results for C_2_H_4_ gas.

The detection limit obtained corresponding to a general integration time of 1s is taken as the lower detection limit of the system. The system was used to measure the standard gas continuously for 30 min, and the data of continuous measurement were evaluated and analysed by using Allan's variance, and the results are shown in Fig. 8, from which it can be seen that the detection limits of the C_2_H_4_ gas concentration under the condition of 14.4 m optical range are 2.85 ppm, respectively, corresponding to the optimal integration time of 56 s. The noise of the system before 56 s is mainly the white noise, and that after 56 s is mainly the noise from system instability factors, such as the drift of the system center frequency, interference fringes and the background spectral line drift. The system noise is mainly the noise of system instability factors, such as the noise caused by the drift of the laser centre frequency, interference fringes and background spectral line drift. The lower detection limit of the system is mainly governed by the system instability, and the Allan variance evaluation results indicate the stability and detection sensitivity of the system. As can be seen from Fig. 8, the lower detection limit can be up to 2.85 ppm at an averaging time of 56 s. Because of the system's own drift, the lower detection limit can not be further lowered by increasing the integration averaging time.

**Figure 9:**
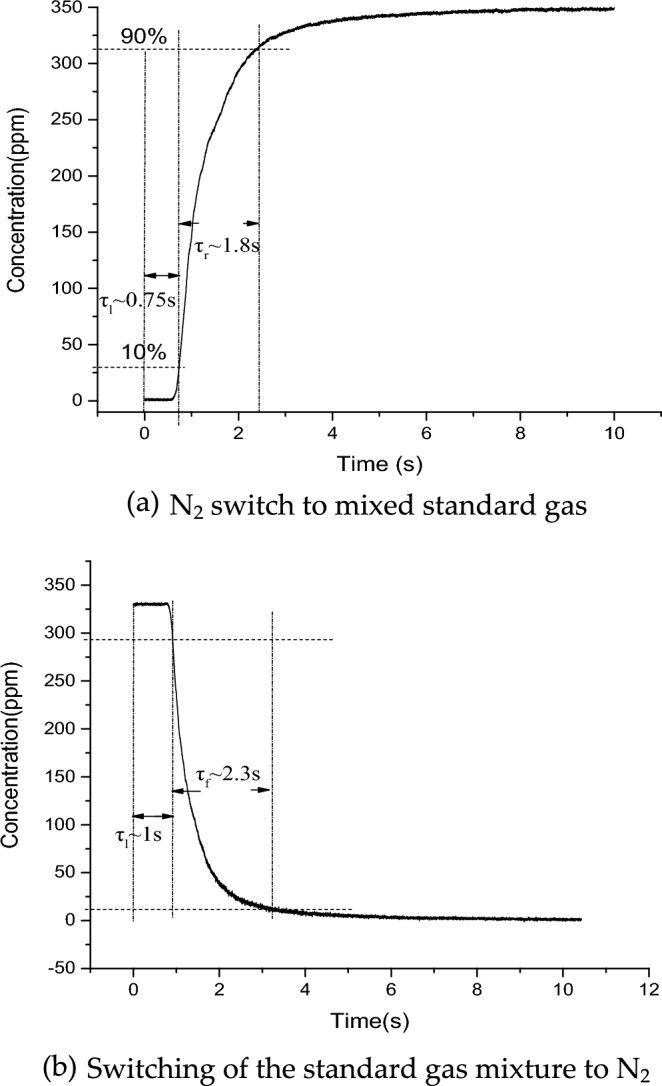
System response curve when switching between mixed standard gas and pure nitrogen gas (**a**) N_2_ switch to mixed standard gas, (**b**) switching of the standard gas mixture to N_2_.

Evaluating the lag time, rise time and fall time of the system, the system response curve when switching between mixed standard gas and pure nitrogen is shown in Fig. 9. From the figure, it can be seen that the rise lag time of the system is about 0.75 s and the fall lag time is about 1s. The rise response time of the system is about 1.8 s and the fall response time is about 2.3 s. The fall response is 28% longer than the rise response time.

**Figure 10:**
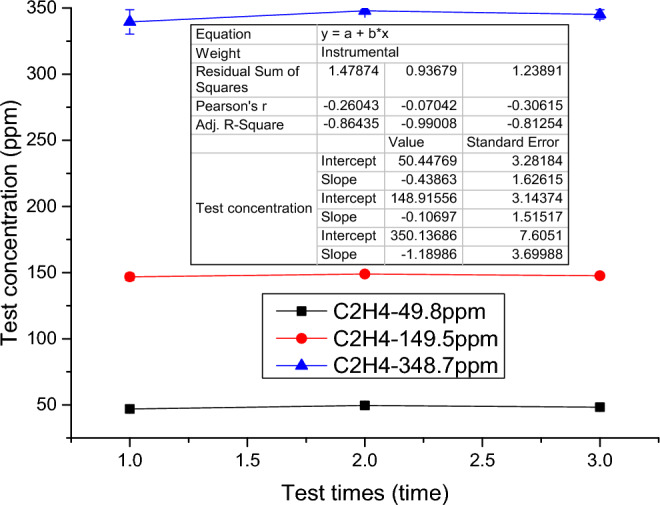
C_2_H_4_ Oscillometric Error Test Results.

The test results are shown in Fig. 10, the absolute error of C_2_H_4_ gas concentration is obtained by calculation, and the relative error with larger absolute value is taken as the indication error, then the indication error of C_2_H_4_ gas concentration is 3.08%, which meets the indicator requirement of indication error <±5.0% in the standard.

In order to improve the speed and accuracy of gas concentration measurement by laser wavelength modulation spectrometry (WMS), a gas concentration measurement method was developed by fitting the main peak of the harmonic signal of the absorption spectrum, in which the peak point of the harmonic signal (WMS 2f/lf) is obtained by scanning only the main peak portion of the harmonic signal of the absorption spectrum. Since the scanning range of the absorption spectrum is reduced, the scanning speed is improved. The measurement accuracy was further improved by fitting a polynomial to the main peak portion of the harmonic signal (WMS 2f/lf) of the absorption spectrum. This method can effectively reduce the effects of random measurement noise, laser power drift and random laser fluctuation, and improve the measurement accuracy while ensuring the measurement speed.

### Algorithm model and steps

For the C_2_H_4_ gas, its concentration Pressure and C can be derived using Eqs. (2) and (3), Temperature T is detected by sensors.

2 $$\Delta {\mathrm{V}}_{\mathrm{C}}=\mathrm{P}\cdot \left[{\mathrm{C}}_{{\mathrm{C}}_{2}{\mathrm{H}}_{4}}\cdot {\upgamma }_{\mathrm{self}}+\left(1-{\mathrm{C}}_{{\mathrm{C}}_{2}{\mathrm{H}}_{4}}\right)\cdot {\upgamma }_{\mathrm{mix}}\right],$$

3 $${\mathrm{C}}_{{\mathrm{C}}_{2}{\mathrm{H}}_{4}}=\frac{\mathrm{A}}{\mathrm{P}\cdot \mathrm{S}(\mathrm{T})\cdot \mathrm{L}},$$

#### Algorithm model

Assuming that the experimentally measured WMS-2f/1f signal was $(v_{i} ,y_{i} ),i = 1,2,3 \ldots n$, with the horizontal axis $v_{i}$ representing the signal frequency, and the vertical axis $y_{i}$ representing the signal amplitude. The nonlinear undetermined parameters in the simulated WMS-2f/1f signal amplitude $S(v)$ included the f absorption line peak frequency $v_{0}$, the Lorentz broadening $\Delta v_{C}$, the Gauss broadening $\Delta v_{D}$ and the integral absorbance $A$. By denoting the simulated WMS-2f/1f signal amplitude as $S(v,v_{0} ,A,\Delta v_{C} ,\Delta v_{D} )$ and the vector $a$ as $(v_{0} ,A,\Delta v_{C} ,\Delta v_{D} )$, the nonlinear least-squares mathematical model fitted by multiline fitting can be expressed as:

4 $$\underset{x\in {R}^{n}}{\mathrm{min}}R(\alpha )=\sum_{i=1}^{i=n}{[{y}_{i}-S({v}_{i},\alpha )]}^{2}$$

To solve the above mathematical model, the Levenberg-Marquardt (L- M) algorithm was used, which gradually approximated the model by addressing a series of linear least-squares problems. Apart from fast convergence, the algo-rithm can also effectively resolve the singular matrix problem during iterative process.

#### Algorithm steps

The steps for simultaneous detection of C_2_H_4_ concentration and pressure by the multiline fitting algorithm are as follows:

*Step 1* Initially, the WMS-2f/1f signals of the four C_2_H_4_ gas absorption lines were fit. In the multiline fitting algorithm, the center frequency of each absorption line was $v_{0 - i}$, the Lorentz broadening was $\Delta v_{C - i}$, and the integral absorbance $A_{i}$. Initial parameters of the Lorentz broadening, which was a free variable of the multiline fitting process, were estimated.

**Figure 11:**
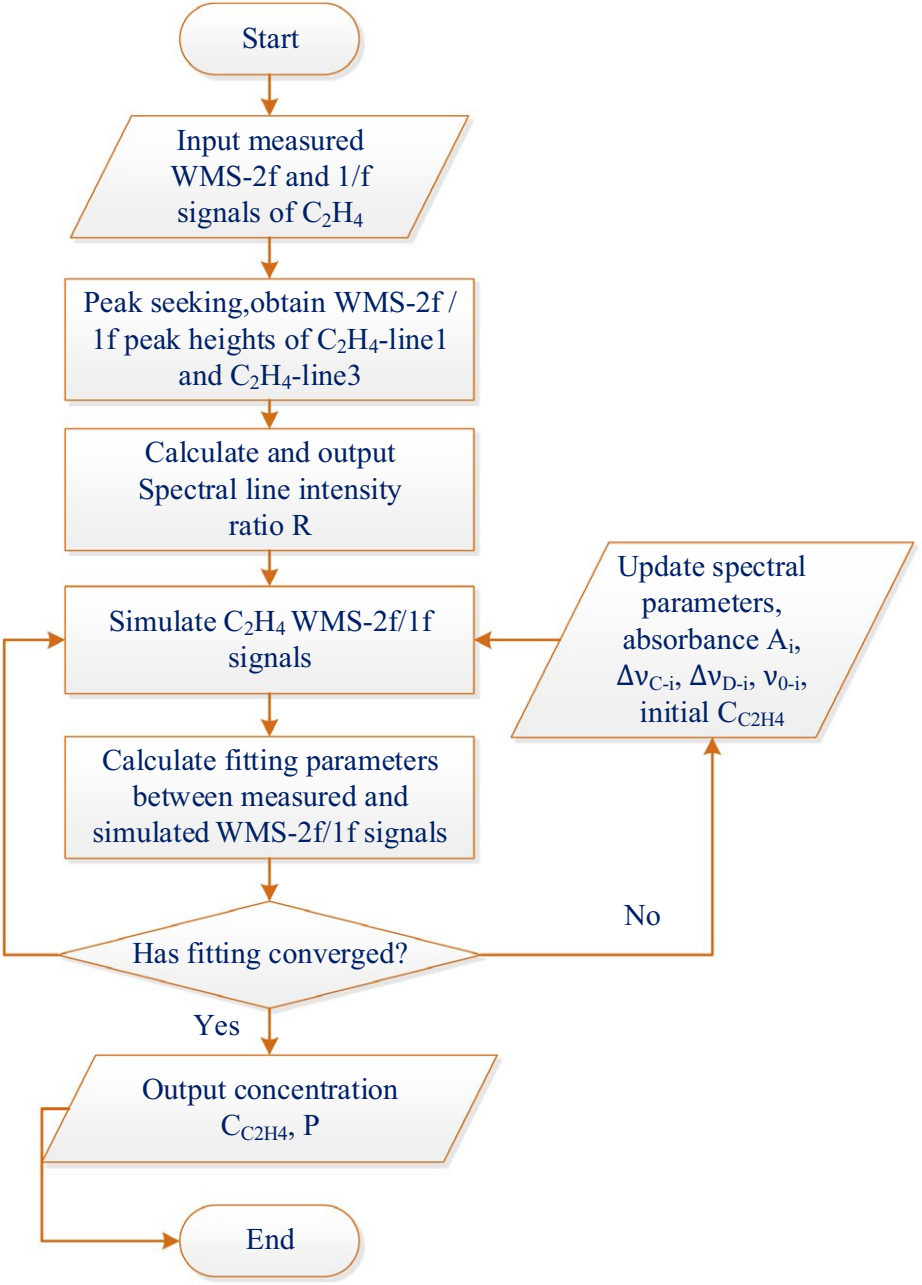
Flowchart of the multiline fitting algorithm.

**Figure 12:**
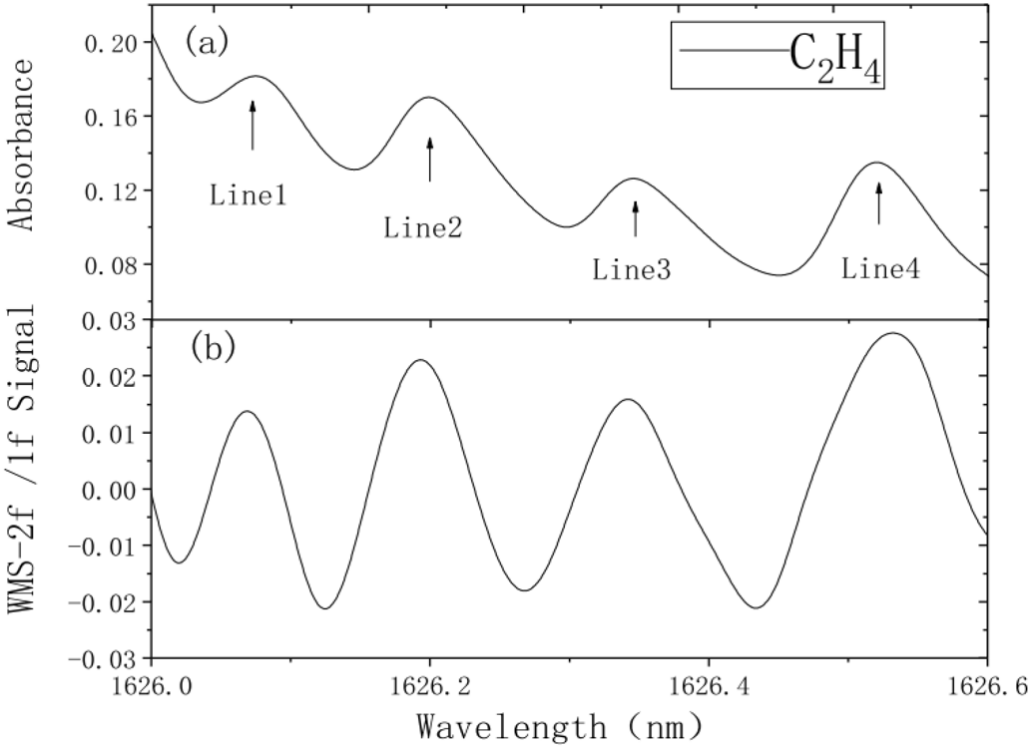
Direct absorption signal and simulated WMS-2f./1f. signal (**a**) Absorbance; (**b**) WMS-2f./1f. Signal).

*Step 2* Every set of free variables in Step 1 had a corresponding WMS-2f/1f signal. Each set of free variables and the measured WMS-2f/1f signals were nonlinearly iterated and, in each iteration, the integral absorbance values $A_{1}$ and $A_{3}$ of the two C_2_H_4_ absorption lines (Fig. 12a), Line 1 and Line 3, could be obtained. Accordingly, $R = A_{1} /A_{3}$ could be derived, which was the line intensity ratio. Based on several update iterations, the formula (4) converged to the minimum, at which the optimal fitting parameters could be obtained. Finally, the concentration and pressure of C_2_H_4_ gas could be inverted using formulas (2) and (3). Figure 11 displays the flowchart of the multiline fitting algorithm.

The multiline fitting algorithm was implemented by Labview software. The mathematical model was fitted by the L-M nonlinear least squares, and through initial parameter estimation of C_2_H_4_ absorption line, the measured and simulated WMS-2f/1f signals were updated and iterated multiple times until the algorithm converged to the minimum, obtaining the optimal fitting parameters. Finally, the C_2_H_4_ concentration and pressure were derivable by inversion calculation.

### Algorithm simulation verification

To confirm the efficiency of the multiline fitting algorithm, simulation analysis was carried out on the algorithm. Using the absorption spectrum information of C_2_H_4_ gas from the PNNL database, the absorbance values and WMS-2f/1f signals of direct absorption spectra were obtained in the wavelength range of 1626–1626.6 nm for C_2_H_4_, as shown in Fig. 12.

**Figure 13:**
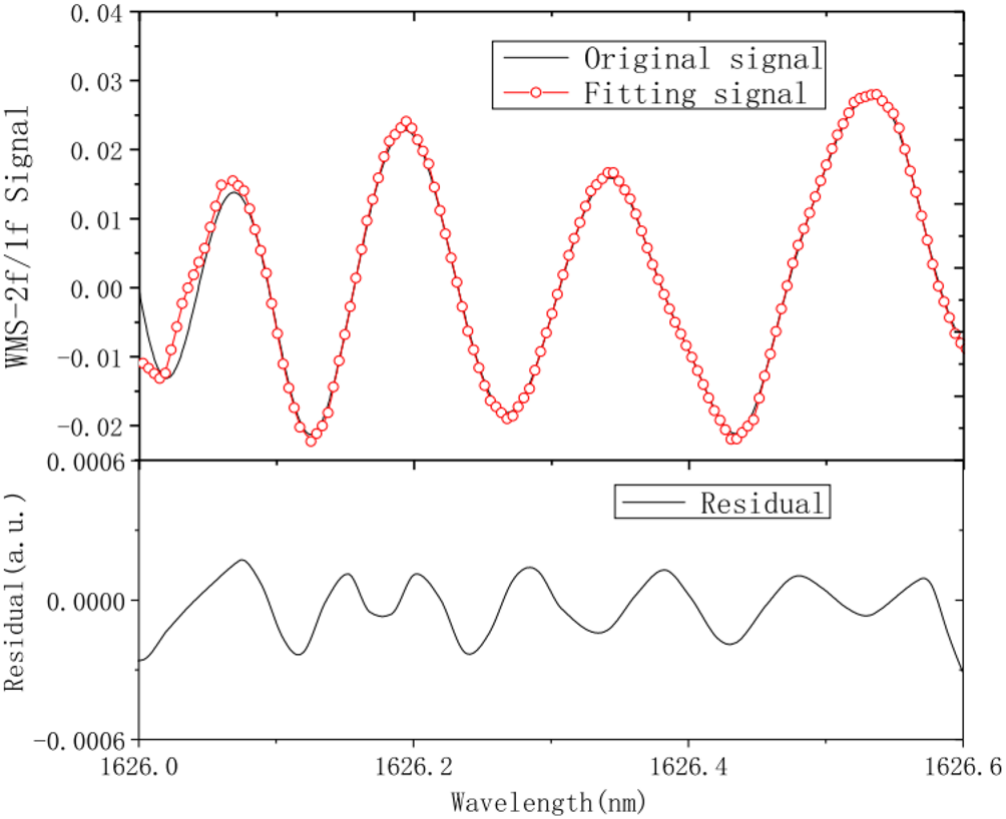
Multiline fitting results.

**Figure 14:**
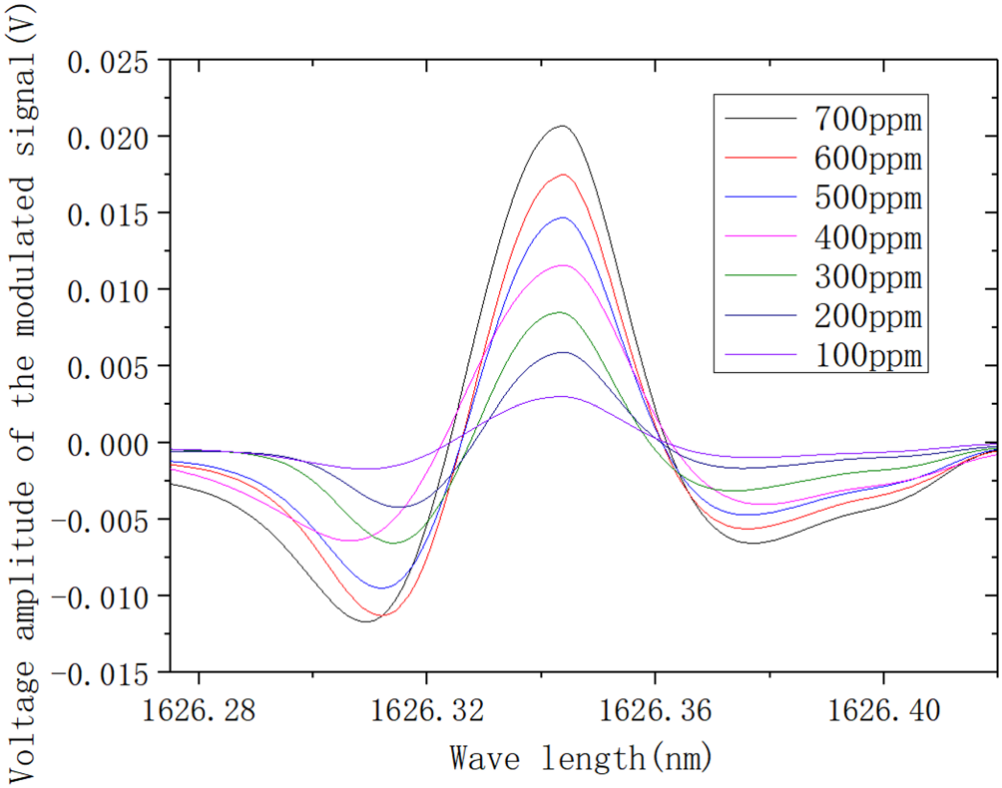
2f. signals at different concentrations.

Using the multiline fitting algorithm, the measured WMS-2f/1f signals of C_2_H_4_ were subjected to multiline fitting through the L-M universal global optimization. At first, the initial parameters were set according to Step 1: the absorption line center frequency of C_2_H_4_ gas was $v_{0}$, the integral absorbance of absorption line was $A$ , the Lorentz half-width was $v_{C}$, the gas pressure was P. The fitting converged quickly, and the results are shown in Fig. 12. The obtained C_2_H_4_ concentration is 689.7 × 10^–6^, which agrees with the initial setting value of 700 × 10^–6^. The goodness of fit between the original and fitted signals is very high, with fitting residuals of less than ± 0.03%. The relatively large residual error at the initial position is caused by the inaccurate acquisition of 2f. signal resulting from the higher current during low-frequency sawtooth wave scanning than the laser threshold current. Figures 12, 13 present a temperature of 296 K, a pressure of 1 atm and information on the C_2_H_4_ gas concentration of 700 ppm (700 × 10^–6^). Figure 14 displays a temperature of 296 K and a pressure of 1 atm.

Using the afore-described multiline fitting algorithm, simulation inversion was performed on the WMS-2f/1f signals with different C_2_H_4_ concentrations, and the results are detailed in Tab. 2. Apparently, the maximum relative error is 2.40%, verifying the feasibility, validity and accuracy of the multiline fitting algorithm in the concentration inversion of hydrocarbon gases with cross-aliasing spectra of absorption lines.

**Table 2:** Multiline fitting inversion results under different C_2_H_4_ concentrations.

Serial number	1	2	3	4	5	6	7
Set value/ × 10^−6^	100	200	300	400	500	600	700
WMS-2f./measured value/ × 10^−6^ WMS-2f./1finversion value/ × 10^−6^ relative error/%	112.6	217.5	319.2	427.3	538.4	649.7	759.6
	102.4	197.5	306.9	397.6	494.3	605.1	707.8
	2.40	1.25	2.30	0.60	1.14	0.85	1.11

### Analysis of second harmonic calibration experimental results

**Figure 15:**
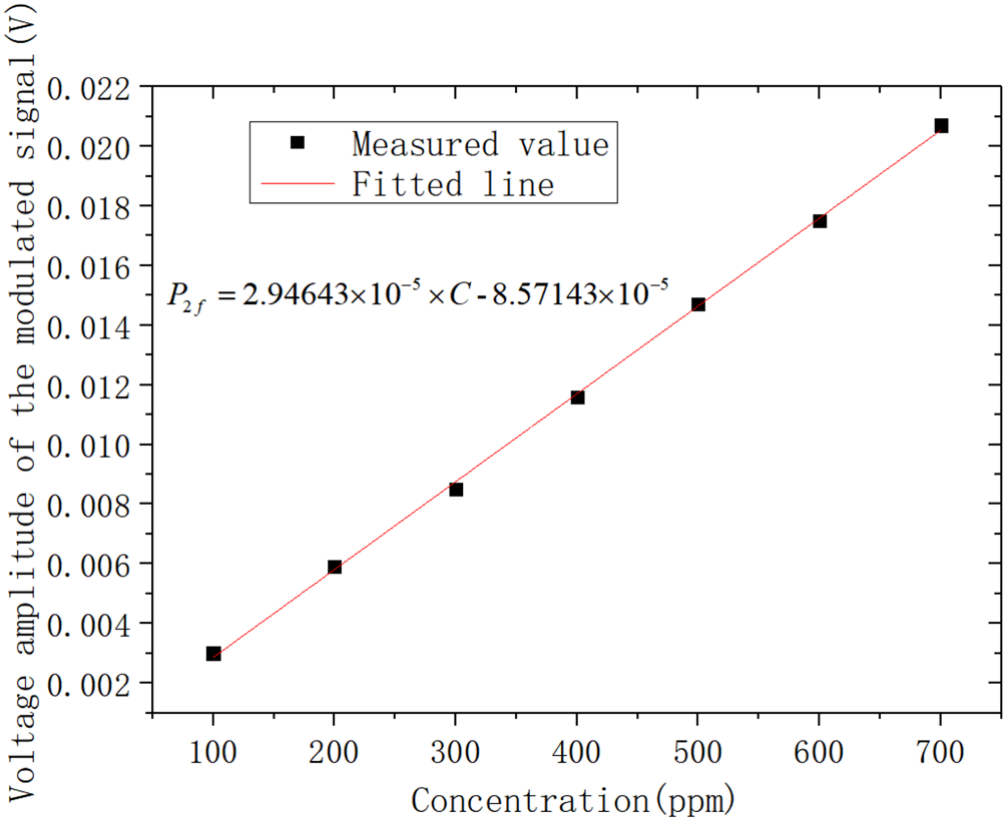
2f. signal peak versus gas concentration fitting curve.

C_2_H_4_ gas at 0.07% was used as the standard gas, and six different concentrations of C_2_H_4_ gas at 0.01%, 0.02%, 0.03%, 0.04%, 0.05% and 0.06% were prepared by Environics 2000 high-precision automatic gas distribution device for calibration experiments. The low-frequency scanning signal, and the high-frequency sine wave signal of 5 kHz were used as the modulating signal. The scanning amplitude was 15 mA, and the modulating signal amplitude was 100 mV. Through the laser current drive with the temperature controller being converted to 2 mA modulating current signal, the sampling rate of the system was 250,000 sampling points per second. The 2f signal was obtained by six calibration experiments with different C_2_H_4_ gas concentrations, as shown in Fig. 14. A linear fit to the peak of the 2f signal at the center wavelength of the absorption spectral line located at 1626.343 nm was performed, and the results are shown in Fig. 15. The fitted relationship between the peak of the second harmonic signal of the absorption spectral line and the C_2_H_4_ gas concentration can be obtained as follows:

5 $${P}_{2f}=2.94643\times {10}^{-5}\times C-8.57143\times {10}^{-5}$$

Table 3 shows the measurement results and errors of different C_2_H_4_ gas concentrations using the WMS-2f method. The absolute error in the calibration of C_2_H_4_ gas concentration using the WMS-2f method is 2.12 × 10^–7^, which is caused by the interference of the C_2_H_4_ gas spectral lines with each other. The detection limit of C_2_H_4_ gas was 2.9 × 10^–7^ at 1626.343 nm With 2f WMS technology, C_2_H_4_ gas is detected with a response time of approx. 6.5 s and a minimum detection limit of 42.60 ppm. With 2f/1f WMS technology, C_2_H_4_ gas is detected with a response time of approx. 1.8s and a minimum detection limit of 2.85 ppm.

**Table 3:** Measurement results and errors of different C_2_H_4_ gas concentrations using the WMS-2f method*.*

Proportional concentration (ppm)	Second harmonic peak/V	Fitting concentration (ppm)	Concentration error/ × 10^–6^
700	0.0207	721.2	21.2
600	0.0175	594.7	− 5.3
500	0.0147	492.4	− 7.6
400	0.0116	397.3	− 2.7
300	0.0085	287.8	− 12.2
200	0.0059	203.9	3.9
100	0.0030	94.6	− 5.4

## Discussion

For the spontaneous coal combustion indicator gases C_2_H_2_, CH_4_ and C_2_H_4_, which are hydrocarbon gases, a cross-interference problem is present between absorption spectral lines. Using multiline fitting algorithm in combination with WMS technique, the measured and simulated WMS-2f/1f signals are subjected to multiline fitting. Based on the extracted spectral line information, the inversion calibration of C_2_H_4_ concentration is achieved. The maximum relative error of C_2_H_4_ concentration measurement is found to be 2.40%, showing a 5.24% reduction compared to that before calibration. Based on the obtained results, the multiline fitting method for gas concentration inversion can effectively eliminate the influence of aliasing interference between absorption spectral lines, which reduces the measurement error of the TDLAS gas detection system, and improves the detection accuracy and stability. Moreover, the proposed method provides a solution for improving the TDLAS-based gas detection performance under multiline cross-interference and offers a reliable technical means for the early warning of spontaneous coal combustion, which has practical significance and promotion value.

## Data Availability

The datasets used and/or analysed during the current study available from the corresponding author on reasonable request.
